# Efficacy and safety of sequential versus quadruple therapy as second-line treatment for helicobacter pylori infection—A randomized controlled trial

**DOI:** 10.1371/journal.pone.0183302

**Published:** 2017-09-28

**Authors:** Daniela Munteanu, Ohad Etzion, Gil Ben-Yakov, Daniel Halperin, Leslie Eidelman, Doron Schwartz, Victor Novack, Naim Abufreha, Pavel Krugliak, Alexander Rozenthal, Nava Gaspar, Alexander Moshkalo, Vitaly Dizingof, Alexander Fich

**Affiliations:** 1 Institute of Gastroenterology and Liver Diseases, Soroka University Medical Center, Faculty of Health Sciences, Ben-Gurion University of the Negev, Beer-Sheva, Israel; 2 Clinical Research Center (CRC), Soroka University Medical Center, Faculty of Health Sciences, Ben-Gurion University of The Negev, Beer-Sheva, Israel; University Hospital Llandough, UNITED KINGDOM

## Abstract

**Background and aims:**

Quadruple therapy is recommended as second-line treatment for *Helicobacter pylori* eradication failure. However, high cost, multiple side effects, and low adherence rates are major drawbacks to its routine use. Our aim was to compare the efficacy and safety of sequential versus quadruple regimens as second line treatment for persistent *Helicobacter pylori* infection.

**Methods:**

Prospective, randomized, open label trial was conducted at a large academic, tertiary care center in Israel. Patients who previously failed a standard triple treatment eradication course were randomly assigned (1:1) to receive a 10-day sequential therapy course, or a 14-day quadruple regimen. Compliance and adverse events were evaluated by telephone questionnaires. The primary endpoint for analysis was the rate of *Helicobacter pylori* eradication as defined by either a negative 13C-urea breath-test, or stool antigen test, 4–16 weeks after treatment assessed under the non-inferiority hypothesis. The trial was terminated prematurely due to low recruitment rates.

See S1 Checklist for CONSORT checklist.

**Results:**

One hundred and one patients were randomized. Per modified intention-to-treat analysis, eradication rate was 49% in the sequential versus 42.5% in the quadruple regimen group (p-value for non-inferiority 0.02). Forty-two (84.0%) versus 33 (64.7%) patients completed treatment in the sequential and quadruple groups respectively (p 0.027). Gastrointestinal side effects were more common in the quadruple regimen group.

**Conclusion:**

Sequential treatment when used as a second line regimen, was non-inferior to the standard of care quadruple regimen in achieving *Helicobacter pylori* eradication, and was associated with better compliance and fewer adverse effects. Both treatment protocols failed to show an adequate eradication rate in the population of Southern Israel.

**Trial registration:**

ClinicalTrials.gov NCT01481844

## Background

*Helicobacter pylori (H*. *pylori*) is known to play a major contributory role in the pathogeneses of chronic gastritis, peptic ulcers, and gastric malignancies[1]. Consequently, great emphasis has been placed on its successful eradication. Many first-line treatments have been employed for this purpose, and the most successful regimen has been reported to achieve eradication rates ranging from 75 to 90%[2]. However, over the last 2 decades or so, failure of *H*. *pylori* eradication has become a common problem in many practice settings. This has mainly been attributed to a rise in *H*. *pylori* resistance to antibiotic therapy seen in many countries throughout the globe, including Israel[3–6]. Another problem that poses a major obstacle to successful treatment is patients’ adherence to antimicrobial treatment regimens. Intolerable side effects with prolonged course of treatment can lead to premature discontinuation of therapy and subsequent failure of bacterial eradication. Furthermore, premature cessation of treatment is a major cause for development of resistant *H*. *pylori* strains[7]. Patients with persistent *H*. *pylori* infection, despite antibiotic therapy, present a greater challenge with respect to successful cure[8]. A number of salvage regimens have been evaluated in patients with persistent *H*. *pylori* infection. Currently, the internationally recommended second line treatment for *H*. *pylori* infection is a quadruple drug regimen (QR) consisting of a proton pump inhibitor (PPI), bismuth salt, metronidazole, and tetracycline for a minimum of 7 days[9, 10]. However, a recent pooled analysis of trials evaluating this regimen, has demonstrated a mean treatment failure rate of nearly 25%.[8] Therefore, a more effective, simplified and better-tolerated drug regimen is greatly needed.

Sequential therapy (ST) is an alternative first-line therapeutic approach for *H*. *Pylori* eradication[11–13]. Use of This regimen involves sequential rather than simultaneous administration of antibiotics, which may decrease treatment-related side effects and improve patient compliance to treatment. This in turn may translate into improved eradication rates in treatment naïve patients. Indeed, in a meta-analysis comparing ST versus standard PPI-based triple therapy as first line treatments for *H*. *Pylori* infection, ST appeared to be superior to triple therapy in achieving *H*. *pylori* eradication[14]

The utility of ST regimens as second-line therapy for resistant *H*. *Pylori* infection has been previously evaluated in only few studies in the literature. However, these were mainly single arm studies with varying drug regimens and treatment length ranging from 10 to 14 days. Furthermore, all but one of these studies employed sequential regimens based on drugs not readily available such as levofloxacin and gatifloxacin. Results from these studies are inconclusive[15–18]. The goal of this randomized controlled trial was to compare the efficacy and tolerability of ST versus QR as second line treatment for *H*. *pylori* infection. We elected to compare regimens comprising drugs readily available in our clinical setting and standard treatment length of 10 and 14 days for ST and QR respectively that were most widely accepted in Israel at the time the study was initiated.

## Methods

### Patient population

This was a prospective, single-center, randomized, open label trial, conducted at Soroka University Medical Center (SUMC), a tertiary care hospital located in southern Israel. We included patients in whom a first eradication trial with triple therapy regimen has failed to eradicate *H*. *Pylori* infection. Patients were considered to have failed a first line regimen if following such treatment, gastric histology showed evidence of *H*. *pylori* organisms, or either rapid urease, *H*. *Pylori* stool antigen or 13C-urea breath test performed within one year before enrollment to this trial remained positive. Patients were excluded if they were < 18 of age, had a past history of gastrectomy, gastric malignancy (including adenocarcinoma and lymphoma), previous allergic reaction to antibiotics (amoxicillin, clarithromycin, metronidazole, and tetracycline) or PPIs, active upper gastrointestinal bleeding within a week prior to enrollment and those with severe concurrent illness or malignancy. In addition we excluded patients who had contraindications to treatment drugs, and pregnant or lactating women.

### Intervention

Patients were enrolled by one of physicians of the Institute of Gastroenterology and Liver Diseases at SUMC, acting as principal or associate investigators on the study. Patients enrolled between January 1^st^ 2012 to June 31^st^ 2015, were randomly assigned (1:1), to receive one of the following two treatment regimens: sequential therapy i.e. 5 days of PPI (lansoprazole 30mg BID) + amoxicillin (1g BID) followed by 5 days of PPI (lansoprazole 30mg BID) + two antimicrobial drugs (clarithromycin (500mg BID) and tinidazole (500mg BID)) or, quadruple drug regimen i.e. 14 days of PPI (lansoprazole 30mg BID) + bismuth (525mg QID) + metronidazole (500mg TID) + tetracycline (500mg QID)/doxycycline (100mg BID)(during the enrollment period tetracycline was changed to doxycycline due to interruption of tetracycline drug supply). Drug adherence and adverse side effects to therapy were assessed via telephone questionnaire 1 week following completion of treatment or pill counting. *H*. *pylori* eradication was defined as a negative ^13^C-urea breath or stool antigen test 4–16 weeks after completion of eradication treatment [17].

### Endpoints

The primary endpoint of this study was the *H*. *Pylori* eradication rate, 4–16 weeks after treatment completion. Secondary endpoints included compliance and adverse events such as: taste alteration, peripheral neuropathy, seizures, nausea, vomiting, diarrhea, abdominal pain, allergic reaction and photosensitivity. Compliance was considered to be satisfactory when the Medication Possession Ratio (MPR), equal to the number of purchased/prescribed daily doses exceeded 80%. Primary analysis included patients who have either completed the protocol or had bacteriological test to confirm eradication, or failed to complete the treatment protocol. For the purposes of the primary analysis we considered patients, who had not completed treatment to have non-eradicated infection (modified intention to treat [ITT]). Per-protocol analysis included patients who had completed the treatment protocol and returned for the bacteriological testing.

### Statistical analysis

Sample size for the trial was calculated based on the following assumptions: estimated proportion of the successful eradication in both groups of 75%, non-inferiority delta of 15%, one sided alpha level of 0.05 and power of 80%. Randomization (1:1) was based on an automated assignment scheme (balanced randomization) generated before the study initiation.

With planned enrollment of 116 evaluable subjects in each arm, we were expecting 90% power to show a difference between assumed compliance rate of 60% in quadruple therapy and 80% in sequential with two-sided alpha level of 0.05. Due to the sequential testing of the hypotheses (efficacy and then compliance) no adjustment of alpha level was required. The trial was terminated after the enrollment of 101 subjects due to the slow recruitment rate. Based on the observed results we have calculated conditional power to accept H0 (sequential therapy *is inferior* to the quadruple therapy).

Continuous variables were compared by t-test and presented as means ± standard deviation. Categorical variables were presented as proportions and compared using Chi-square and Fischer exact tests. The data was summarized using frequency tables, summary statistics, confidence intervals and p-values as appropriate. Gart-Nam score was used for the non-inferiority testing [19]. Two sided p-value <0.05 was considered to be statistically significant unless specified otherwise.

All statistics were performed using SPSS version 20 (SPSS Inc. Chicago, Illinois)

## Ethics approval and consent to participate

The study was approved by the SUMC Institutional Review Board and conducted according the principles of the declaration of Helsinki and Good Clinical Practice guidelines. All study participants gave written informed consent prior to enrollment. See S1 File for study protocol.

## Results

**Fig 1:**
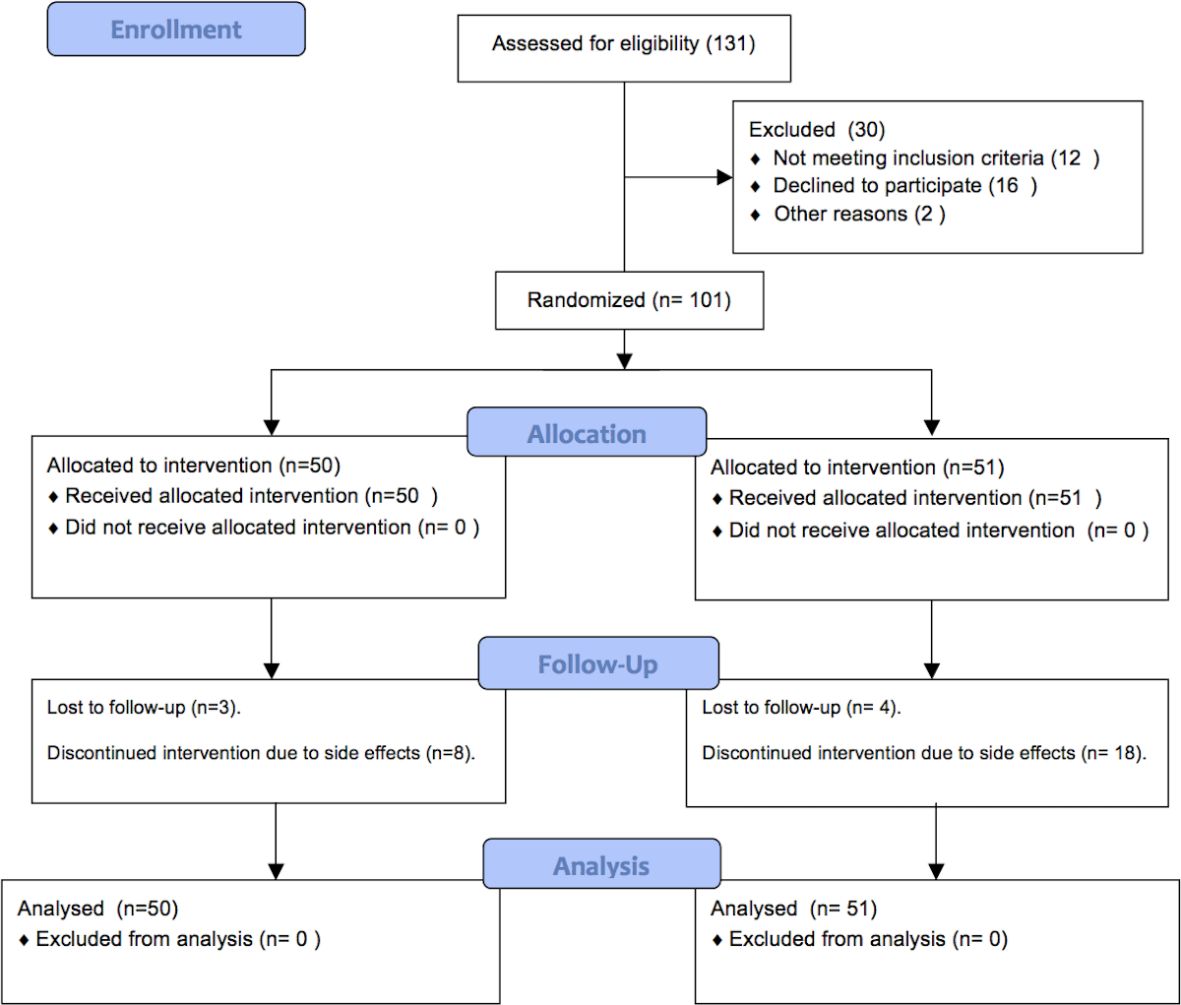
CONSORT flow diagram.

**Fig 2:**
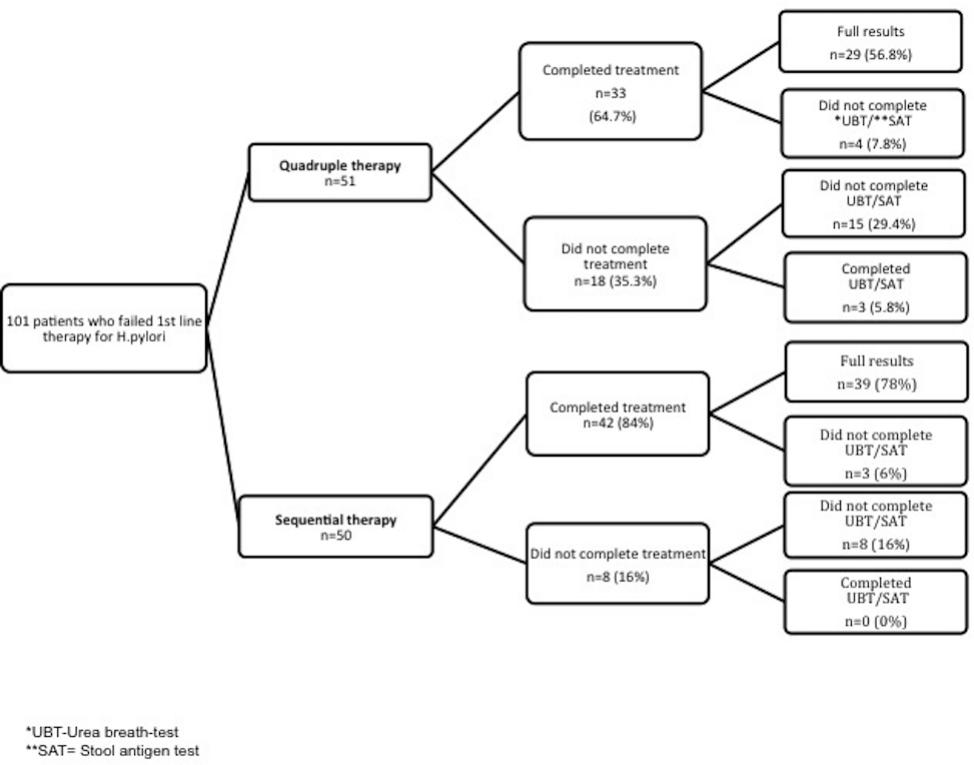
Study flow chart.

The CONSORT flow diagram and study flow chart are depicted in Fig 1 and Fig 2 respectively. A total of 101 patients from the SUMC Gastroenterology clinic were randomized to receive either ST (50 patients) or QR (51 patients), between January 1^st^ 2012 to June 31^st^ 2015. The baseline demographic and clinical characteristics of patients in this study are listed in Table 1. Mean age (43 in both groups), gender distribution (35–40% male) and comorbidities were similar between the 2 arms.

**Table 1:** Patient baseline characteristics.

		**Sequential (n = 50)	*Quadruple (n = 51)
Age (mean ± SD)		43.94 ± 15.75	43.75 ± 17.08
Gender	Male (n, %)	20 (40%)	18 (35.3%)
**Comorbidities**			
Family history of gastric cancer (n, %)		1 (2%)	5 (9.8%)
Alcohol or drug abuser (n, %)		2 (4%)	1 (2%)
Anemia (n, %)		11 (22%)	12 (24%)
Smoker (n, %)		9 (18%)	4 (8%)
Diabetes (n, %)		1 (2%)	7 (13.7%)
**Chronic medications**			
Aspirin (n, %)		3 (6%)	6 (12%)
Anticoagulation (n, %)		1 (2%)	1 (2%)
Other medications (n, %)		19 (38%)	20 (40%)

* The quadruple therapy is the recommended second line of treatment for *H*. *pylori* infection and includes 14 days of PPI+ bismuth + metronidazole + tetracycline/doxycycline.

** The ST regimen includes 5 days of PPI + amoxicillin followed by 5 days of PPI + two antimicrobial drugs (clarithromycin and tinidazole).

Table 2 shows previous *H*. *pylori* treatment and modality used for diagnosis of the current infection. Median time elapsed from the previous first line QR was six month (IQR 4–14 months), while most of the patients (91%), were treated with the standard triple therapy regimen (amoxicillin, clarithromycin, PPI).

**Table 2:** Previous H. pylori treatment and current infection diagnosis.

		Sequential (n = 50)	Quadruple (n = 51)
Previous treatment recommended by (n, %)	Family physician	42 (84%)	39 (78%)
	Gastroenterologist	8 (16%)	11 (22%)
	Other	0	1
Date of last triple therapy (months) * Median (IQR)		6 (4–14.25)	6 (4–12)
Amoxicillin + Clarithromycin (n, %)		47(94%)	45 (91.8%)
Amoxicillin + flagyl (n, %)		4 (8%)	2 (4.1%)
Other (n, %)		0 (0%)	4 (7.8%)
**Infectious status verification** *			
Histology (+) (n, %)		4 (8%)	5 (10%)
CU-test (+) (n, %)		11 (22%)	11 (22%)
Urease breath test (+) (n, %)		9 (18%)	17 (34%)
Stool Ag (+) (n, %)		31 (62%)	24 (48%)

*Percentage can add up to more than 100% due to multiple treatments attempts

Table 3 depicts clinical outcomes and compliance analysis. Out of 50 patients in the ST group, 42 (84%) completed treatment. Eradication rate in modified ITT population, comprising patients who have completed treatment and whose infection status was known, and those who have not completed treatment (presumed to be infected), was 49.0% (23/47). In those who completed the treatment protocol and performed the follow-up test (per protocol analysis), eradication rate was 59.0% (23/39).

**Table 3:** Comparison of outcomes between the two treatments arms.

	Sequential (n = 50)	Quadruple (n = 51)	p-value
Completed treatment protocol sequential/quadruple (n, %)	42/50 (84.0%)	33/51 (64.7%)	0.027
Completed protocol and did not preform bacteriological follow-up test (n/m, %)^1^	3/42	4/33	0.69
Treatment success (eradication), modified ITT analysis^2^	23/47 (49.0%)	20/47 (42.5%)	0.53
Treatment success (eradication), per-protocol analysis^3^	23/39 (58.9%)	20/29 (68.9%)	0.39

1 Completed treatment administration but did not preformed the urea/stool test thus consider a failure of treatment.

2 Primary analysis included patients who had either urea breath test to confirm eradication or failed to complete the treatment protocol. For the purposes of the primary analysis we considered patients, who had not completed treatment to have non-eradicated infection.

3 Per-protocol analysis included patients who had completed the treatment protocol and returned for bacteriological testing.

In the QR group, only 33 patients (64.7%) completed treatment, of which 29 returned to follow up and performed urea breath test or stool examination. Eradication rate in the modified ITT analysis in this population was 42.5% (20/47). Therefore the difference between the eradication rates in the sequential and quadruple groups was 6.4% (95% CI -14.0% to 27.0%) and p-value for non-inferiority was 0.02.

Compliance to treatment was significantly better in the sequential vs. quadruple treatment arms (p = 0.027).

Table 4 depicts rates of side effects between the two treatments. Gastrointestinal side effects were reported by 27 (65.9%) subjects in the QR group as compared to 19 (43.2%) subjects in the ST group (p = 0.036). Patient global assessment showed that 64.3% vs. 51.2% of patients in the sequential and quadruple treatment groups respectively, described their general sense of wellbeing during treatment as either good or unchanged.

**Table 4:** Comparison of side effect profile between the 2 regimens.

	Sequential (n = 50)	Quadruple (n = 51)	p-value
Any GI (n, %)	19 (43.2%)	27 (65.9%)	0.036
Taste alterations (n, %)	5 (11.4%)	10 (24.4%)	0.11
Nausea (n, %)	9 (20.5%)	15 (36.6%)	0.09
Vomiting (n, %)	2 (4.5%)	4 (9.8%)	0.42
Diarrhea (n, %)	1 (2.3%)	3 (7.3%)	0.35
Abdominal pain (n, %)	10 (22.7%)	13 (31.7%)	0.46
Peripheral neuropathy (n, %)	1 (2.3%)	1 (2.4%)	1.0
Other (n, %)	16 (36.4%)	19 (46.3%)	0.35
General feeling during treatment:			
• Bad	15 (35.7%)	19 (48.7%)	0.49
• Good	14 (33.3%)	10 (25.6%)	
• Not changed	13 (31.0%)	10 (25.6%)	

Table 5 shows comparison of cohort characteristics between patients who completed (n = 75) and those who did not complete (n = 26) the assigned treatment. These two groups appeared to be similar with the exception of male gender, which trended towards statistically significant association with a higher rate of therapy completion (p = 0.076).

**Table 5:** Comparison of cohort characteristics by completion of treatment*.

		Complete (n = 75)	Not complete (n = 26)	p-value
Age (mean ± SD)		45.57 ± 16.66	38.85 ± 14.6	0.71
Gender	Male (n, %)	32 (42.7%)	6 (23.1%)	0.076
**Co-morbidities**				
Family history of gastric cancer (n, %)		4 (5.3%)	2 (7.7%)	0.64
Alcohol or drug abuser (n, %)		2 (2.7%)	1 (4%)	1.0
Anemia (n, %)		19 (25.3%)	4 (16%)	0.33
Smoker (n, %)		8 (10.7%)	5 (20%)	0.23
Diabetes (n, %)		6 (8%)	2 (7.7%)	0.96
**Chronic medications**				
Aspirin (n, %)		8 (10.7%)	1 (4%)	0.31
Anticoagulation (n, %)		1 (1.3%)	1 (4%)	0.40
Other medications (n, %)		30 (40%)	9 (36%)	0.72

*Completion of treatment protocol was considered satisfactory when the Medication Possession Ratio (MPR), equal to the number of purchased/prescribed daily doses exceeded 80%. Completion of bacteriological analysis was not defined as criteria for completion of treatment protocol. It includes 42 and 33 patients (total of 75 patients)on sequential and quadruple regimens, respectively.

## Discussion

In this randomized, open label trial, the efficacy of ST was found to be comparable to that of standard of care QR, when given as second line treatment for *H*. *pylori* infection. Furthermore, patients in the ST arm had fewer side effects and showed better compliance to treatment than patients who received QR. The efficacy of ST as first line treatment for *H*. *pylori* infection has been established in multiple studies published over the last decade[11–13,20], however, data supporting the use of ST as an alternative strategy to QR or other second line treatments is scant. The few studies evaluating this approach were mostly single arm prospective trials that showed inconsistent results[15–18]. Recently, Liou et al reported the results of a multicenter randomized controlled trial comparing the efficacy of levofloxacin-based ST vs. triple therapy in patients who failed to eradicate *H*. *pylori* with first line therapies[21]. In this study, ST was shown to be superior to triple therapy in both the ITT and per protocol analysis. Furthermore, the efficacy of ST regimen not only exceeded that of triple therapy in patients infected with levofloxacin sensitive strains, but also in those with levofloxacin resistance bacteria. In the present study different regimens were used for both the sequential and standard treatment arms. Nevertheless, similar to the findings of Liou et al, response rate in the ITT analysis was higher in ST vs. standard treatment arm (although not reaching statistical significance). Interestingly, these results were obtained despite the fact that the ST regimen included an antibiotic (clarithromycin) that was previously used as a first line agent in 94% of patients (Table 2), and for which high rates of resistance have been previously reported in Israel and other countries[3–6]. These results further support Liou et al’s findings and suggests that the beneficial effect of ST on antibiotic resistant *H*. *pylori* strains is probably related to sequential drug administration rather then to the specific type of antibiotics included in the regimen.

The added benefit of ST may be explained by several factors, including the relative ease of administration, improved side effect profile and lower cost of sequential therapy. These factors could have significantly impacted patient compliance to therapy leading to an improved response rate. Indeed, significantly lower rates of treatment completion were recorded in the quadruple versus sequential treatment arms (64.7 vs. 84% respectively; p = 0.027), while fewer patients in the sequential vs. quadruple treatment arm reported GI side effects (43.2 vs. 65.9%; p = 0.036).

Of concern, both sequential and quadruple therapy failed to show an appropriate eradication rate in modified ITT population (sequential-49%, quadruple-42.5%). These results may be explained by relatively high resistance rates to antibiotics that have previously been described in our region[4, 6] and a longer interval between first and second line therapy[22]. Additional factors that may have contributed to the relatively low eradication rates in this study are the employment of a 10 rather than 14-day course of ST (the most widely excepted ST regimen in Israel at the time) and the use of first generation PPI (drugs covered under the Israeli Health Basket) at standard doses. Inadequate gastric acid suppression and treatment length might have lead to the low eradication rate of 10-day sequential therapy in the second line treatment. Future studies are needed to assess the use of new generation or higher dosage of PPI and extending the treatment length of sequential therapy to 14 days [23–25]. Furthermore, disturbing findings from our study call for adoption of alternative strategies for management of patients failing first-line therapies. These may include drug resistance testing prior to initiation of rescue therapies, and use of personally tailored drug regimens based upon results of susceptibility testing [26, 27].

There are several limitations to our study. First, an insufficient number of enrolled patients -101 (as a compared to planned 232 evaluable patients) does not allow exclusion of type I error. However, given the observed rates of eradication that were numerically higher in the ST arm, the conditional power to show the inferiority of the sequential therapy had we enrolled the proposed sample is less than 5%. Second, the trial was conducted as an open label study, thus we cannot exclude that both patients and physicians were influenced by the knowledge of the assigned treatment regimen in their decisions. Third, we used heterogeneous tests to confirm eradication status in this study. While all tests used are widely accepted and were performed in certified labs and according to the manufacturers’ instructions, we cannot exclude that this may have affected the results of the study. Finally, we did not conduct susceptibility testing prior to enrollment to the study. Therefore, the impact of resistant strains to the overall poor response rate to therapy in our cohort, as well as to the comparable response rates between the two arms could not be reliably determined.

In conclusion, ST showed a similar eradication rate as standard of care, second-line treatment for *H*. *pylori* infection, but was associated with better compliance and improved tolerability. Both treatment protocols failed to show an adequate eradication rate in a cohort representing the population of Southern Israel. Further work should be directed at establishing a local susceptibility patterns and eradication rates, and tailoring personalized, culture-guided rescue regimens for patients who have failed first-line therapies for *H*. *pylori* infection.

## Supporting information

S1 Checklist CONSORT 2010 checklist. (DOCX)

S1 File Study protocol. (DOC)

## Abbreviations

H.: *pylori*- Helicobacter pylori
QR: quadruple drug regimen
PPI: proton pump inhibitor
ST: Sequential therapy
SUMC: Soroka University Medical Center
MPR: Medication Possession Ratio
ITT: intention to treat
