# Supplementary material for: Efficacy and safety of sequential versus quadruple therapy as second-line treatment for helicobacter pylori infection—A randomized controlled trial
Source: PLoS One. 2017 Sep 28;12(9):e0183302. doi: 10.1371/journal.pone.0183302 (PMC5619725; doi:10.1371/journal.pone.0183302)
Supplement: S1 File — (DOC) [file pone.0183302.s002.doc]

**Sequential versus quadruple therapy as second- line treatment after failure of standard therapy for Helicobacter pylori infection**

Study protocol

Protocol summary

| 1. PPI( Lansoprazole 30mg x2/day) + amoxicillin 1gx2/day followed by 5 days of PPI(Lansoprazole 30mg x2/day) + two antimicrobial drugs ( Clarithromycin500mg x2/day and Tinidazole 500mg x2/day )  2. quadruple drug regimen (i.e.-14 days of PPI (Lansoprazole 30mg x2/day) + Bismuth Subsalicilate 525mg X4/day + Metronidazole 500mg x3/day + Tetracycline 500mg x4/day or Doxyllin 100mg x2/day ) | Regimens |
| --- | --- |
| Both treatments are approved for the eradication of Helicobacter Pylori | Regulatory status |
| Prospective, randomized, open label trial | Study design |
| The objective is to evaluate the efficacy of sequential therapy, i.e. 5 days of PPI + amoxicillin followed by 5 days of PPI + two antimicrobial drugs( clarithromycin and tinidazole ) versus quadruple drug regimen( i.e.-14 days of PPI+ bismuth + metronidazole + tetracycline ) as second line treatment for Helicobacter pylori eradication | Objective |
| From world experience successful eradication on second line treatment with quadruple therapy is approximately 75 %. We assume a non-inferior eradication rate with second line therapy based on sequential therapy.  In addition we assume better compliance rate (defined as the use of >80% of the prescribed medications) in patients randomized to sequential therapy. | Study hypotheses |
| We are planning to include 258 patients of The Department of Gastroenterology, Soroka Medical Center, and Beer-Sheva, Israel. The study will include consecutive ambulatory patients assessed at Gastroenterological Department, Soroka and family practitioner, who failed standard first line therapy-triple therapy of H.pylori and have indications for eradication of H. pylori. Patients are considered initial treatment failures if histology, rapid urease, 13C-urea breath testing or stool AG test will remain positive despite first-line PPI-based triple therapy. These subjects will be randomized at a 1:1 ratio, by blocks of four. | Number of subjects |
| The primary end points of the study are:   - The eradication rate after second line treatment based on sequential therapy comparative to eradication rate after second line therapy based on quadriple regimen. H.pylori eradication is defined as a negative C-urea breath test beginning 4 weeks after eradication treatment completion. - Rate of compliance to the prescribed therapy (more than 80% of medications used for all medications) | Primary endpoints and measurement |
| Study follow-up period will be 8 weeks. During these 8 weeks the study subjects will be assessed 3 times. | Study follow up |
| The secondary end points:  Adverse effects of sequential and quadruple treatment (taste alteration , peripheral neuropathy, seizures, nausea, vomiting, diarrhea, abdominal pain, allergic reaction, photo sensibility) | Secondary endpoints |
| The sample size calculations will be performed based on the following assumptions:  **Efficacy:**  -Rate of successfully achieved eradication of H.pylori for the standard quadruple therapy is 75%  -Non-inferiority delta of 15%  -Alpha 0.05(one-sided)  -Power 80%  Group sample sizes of 116 and 116 achieve 80% power to detect a statistically significant difference in achieving eradication of H.pylori following the recurrent infection. To accommodate a 10% rate of lost to follow-up we will enroll 258 subjects  **Compliance:**   - In standard therapy group compliance rate is expected to be 60% - We anticipate that in sequential therapy group the compliance rate will be 80%.   With two-sided alpha of 0.05 we have 89% power to detect a significant difference. | Sample size consideration |
| 1.signed informed consent  2.age at least 18 years  3. persisting *H.pylori* infection after at least one course of first-line standard triple therapy (Moxypen, Clarin or Flagyl based) | inclusion criteria |
| 1) history of gastrectomy  2) gastric malignancy, including adenocarcinoma and lymphoma  3) previous allergic reaction to antibiotics (amoxicillin, clarithromycin, metronidazole, tetracycline) and proton pump inhibitors  4) Active upper gastrointestinal bleeding within the previous 1 week  5) contraindications to the treatment drugs  6) Pregnant or lactating women  7) Severe concurrent disease or malignancy | Exclusion criteria |
| Daniela Munteanu, MD , Gastroenterology Department, Soroka Medical Center | Principal study investigator |
| Soroka Clinical Research Center  Head: Victor Novack MD PhD  Email: victorno@clalit.org.il | Data management and analysis |
|  | Study monitor |
| Soroka Medical Center, Beer Sheva, Israel | Study site |
| 07/2011  The enrollment is planned to continue for 18 months | Estimated start data and enrollment phase |
|  |  |

Background

Helicobacter pylori s known to play a major contributory role in the pathogeneses of chronic gastritis, peptic ulcers, and gastric malignancies (1). Consequently, great emphasis has been placed on its successful eradication. Many first-line treatments have been employed for this purpose, and the most successful regimens have achieved eradication rates of 75–90% [[2](http://www3.interscience.wiley.com/cgi-bin/fulltext/118536283/main.html,ftx_abs#b2%23b2)].in Israel- in period of 2003-2007- eradication rate-from different sources- was between 57.5-82 %.(18) Treatment of *H pylori* infection is a common problem in many practice settings. The prevalence of *H. pylori* infection has been falling in developed countries bud difficulties with eradication have been increasing as the prevalence of resistant strains of *H.pylori* increase. In Israel during the period of 2002-2006 rate of resistance rise- for metronidazole- 31.4% to 60%-(18)' for clarithromycin- resistance rare in the same period is -8% -65%.(18). Another major problem with antimicrobial treatment regimens for *H. pylori* is adherence with the treatment regimen. Intolerable side-effects with prolonged course of treatment can lead to premature discontinuation of therapy and the development of resistant strains of *H.pylori*. (19). Patients with persistent H. pylori infection, despite antibiotic therapy, present a greater challenge with respect to successful cure [[3](http://www3.interscience.wiley.com/cgi-bin/fulltext/118536283/main.html,ftx_abs#b3%23b3)].

A number of salvage regimens have been evaluated in patients with persistent H. pylori infection. Currently, the internationally recommended salvage therapy for H. pylori infection is a bismuth-based quadruple drug regimen consisting of a proton pump inhibitor (PPI), bismuth salt, metronidazole, and tetracycline for a minimum of 7 days [[4,5](http://www3.interscience.wiley.com/cgi-bin/fulltext/118536283/main.html,ftx_abs#b4%23b4)]. However, a recent pooled analysis of trials conducted to evaluate this regimen as a salvage therapy for persistent H. pylori infection demonstrated a mean treatment failure rate of nearly 25%, and this was substantially higher in some countries [[3, 6](http://www3.interscience.wiley.com/cgi-bin/fulltext/118536283/main.html,ftx_abs#b3%23b3)]. Major causes of bismuth-based quadruple therapy included antibiotic resistance and noncompliance [[7](http://www3.interscience.wiley.com/cgi-bin/fulltext/118536283/main.html,ftx_abs#b7%23b7)]. Poor patient compliance with quadruple therapy is mainly due to its considerable side-effect rates and complicated dosing schedules [[8](http://www3.interscience.wiley.com/cgi-bin/fulltext/118536283/main.html,ftx_abs#b8%23b8)]. Bacterial resistance to metronidazole is also an important element of treatment failure [[7,9–11](http://www3.interscience.wiley.com/cgi-bin/fulltext/118536283/main.html,ftx_abs#b7%23b7)], and primary resistance to metronidazole is relatively common [[12,13](http://www3.interscience.wiley.com/cgi-bin/fulltext/118536283/main.html,ftx_abs#b12%23b12)] and has a negative impact on the effectiveness of quadruple therapy [[7](http://www3.interscience.wiley.com/cgi-bin/fulltext/118536283/main.html,ftx_abs#b7%23b7)]. For this reason, more effective, straightforward, better tolerated alternative regimens are needed. Furthermore, a rapid increase in resistance to clarithromycin [[13](http://www3.interscience.wiley.com/cgi-bin/fulltext/118536283/main.html,ftx_abs#b13%23b13)] has increased the need to establish a more efficient rescue therapy.

A meta- analysis of studies comparing sequential therapy with standard PPI-based triple therapy as first line treatment showed that the eradication rate was higher with sequential therapy. (20) Sequential therapy is a new concept in eradication therapy. With this form of therapy, antibiotics are administered in a sequence rather than all together. The sequential regimen that has been well described is a 10-day treatment consisting of a PPI and **AMOXYCILLIN** 1G (both twice daily) administered for the first 5 days followed by triple therapy, consisting of a PPI, clarithromycin 500mg , and tinidazole 500 mg (all twice daily) for the remaining 5 days . The exact mechanism by which ST (sequential therapy) works is uncertain. Several possibilities exist but all remain unproven at this time. One possibility is that decreasing the bacterial density in the stomach with a drug such as amoxicillin (to which resistance is rare) with the initial 5 days of therapy, improves the efficacy of the subsequently administered combination of clarithromycin and tinidazole. It is known that bacteria can develop efflux channels for clarithromycin, which rapidly transfer the drug out of the bacterial cell, preventing binding of the antibiotic to the ribosome (21). Therefore, another possibility is that amoxicillin acts on the bacterial cell wall and weakens it in the initial phase of treatment thereby preventing the development of efflux channels by weakening the cell wall of the bacterium. A final possibility is that the ST has more advantage due to the number of anti-microbial to which the organism is exposed.

The aim of this study is to evaluate the efficacy of sequential therapy, i.e. 5 days of PPI + amoxicillin followed by 5 days of PPI + two antimicrobial drugs( clarithromycin and tinidazole ) versus quadruple drug regimen( i.e.-14 days of PPI+ bismuth + metronidazole + tetracycline ) as second line treatment of H. pylori . Currently there is none or few date of second line treatment with sequential therapy. The eradication rate of first line (triple) therapy In Israel is70-75 %. We have no data about second line eradication rate in Israel. From world experience successful eradication on second line treatment with quadruple therapy is approximately 70%, we assume to obtain the same or higher eradication rate with second line therapy based on sequential therapy.

Methods

Study objectives:

Primary objectives of this study is to compare safety and effectiveness of the eradication rate after second line treatment based on sequential therapy comparative to eradication rate after second line therapy based on quadruple regimen .

Primary endpoint: H. pylori negative breath test starting 4 weeks after the completion of the treatment.

The secondary end points are the adverse effects of sequential and quadruple treatment (taste alteration, peripheral neuropathy, seizures, nausea, vomiting, diarrhea, abdominal pain, allergic reaction, and photosensitivity) and patient compliance with it. Compliance is considered to be satisfactory when drug intake exceeded 80%.

We are planning to include 258 patients of The Department of Gastroenterology, Soroka Medical Center, Beer-Sheva, Israel. Also we will include at ambulatory patients - with direct referral from family physician or newspaper publicity .

Inclusion criteria are:

1) Age at least 18 years

2) Persisting *H. pylori* infection after at least one course of first-line PPI–Amoxicillin–Clarithromycin or Metronidazole therapy. Patients are considered initial treatment failures if histology, rapid urease, stool Ag or 13C-urea breath testing will remain positive despite first-line PPI-based triple therapy. These tests should be performed during the one year before enrollment in this study.

Exclusion criteria:

1) Age< 18

2) History of gastrectomy

3) Gastric malignancy, including adenocarcinoma and lymphoma

4) Previous allergic reaction to antibiotics (amoxicillin, clarithromycin, metronidazole, and tetracycline) and proton pump inhibitors

5) Active upper gastrointestinal bleeding within the previous 1 week

6) Contraindications to the treatment drugs

7) Pregnant or lactating women

8) Severe concurrent disease or malignancy.

Written informed consent will be obtained from all patients prior to enrolment.

The 258 patients enrolled will be randomized to receive one of the following two treatment regimens for10/ 14 days the patients will be informed of the importance of full compliance, warned of side-effects, instructed to complete treatment, and provided with a contact number, in case of problems. One week after completing these therapies, compliance will be evaluated by a physician during the visit in clinic by direct questioning or pill counting. Compliance is considered to be satisfactory when drug intake exceeded 80%. In addition, at this time all patients will be interviewed for adverse events. *H. pylori* eradication is defined as a negative ^13^C-urea breath 4-16 weeks after eradication treatment completion [[17](http://www3.interscience.wiley.com/cgi-bin/fulltext/118536283/main.html,ftx_abs#b17%23b17)]. (C-urea breath test- Patients are fasted for 4 h before testing. No test meal will be given, and a pre-dose breath sample is obtained. 75 mg of ^13^C-urea powder dissolved in 50 mL of water is then administered orally, and a second breath sample is collected 30 minutes later. Collected samples are analyzed using an isotope ratio mass spectrometer). The patient should stop receiving PPI for 10 days before the breath test and not receive any antibiotics for 7days before this exam.

Subject screening and treatment flowchart

Ambulatory patients who failed standard triple first line therapy of H.pylori

Obtain informed consent

Enroll and randomize subjects in two Strata:

1-standard quadruple therapy

2- Sequential therapy

Following drug treatment

For standard quadruple therapy-14 days

For sequential therapy-10 days

First evaluation at one week after completing these therapies

Visit in ambulatory clinic and check

Compliance with treatment and reports of adverse effects

Compliance> 80%-

satisfactory

Compliance < 80% -

Unsatisfactory

Second evaluation -8 weeks after completing treatment

C-urea breath test check the results of

Schedule of events

|  | On enrollment | 7 days after completing treatment –visit in clinic | 8 weeks after completing treatment |
| --- | --- | --- | --- |
| Sign informed consent | X |  |  |
| Check inclusion/exclusion criteria | X |  |  |
| Start one of two treatment regimen  ( sequential or quadruple) | X |  |  |
| Check compliance |  | X |  |
| Ask about adverse effects |  | X |  |
| c-urea breath test |  |  | X |
|  |  |  |  |
|  |  |  |  |

**Screening Procedures**

Prior to subject participation in this study, the Investigator must obtain written Ethics Committee (EC) and other local regulatory bodies as appropriate approval for the protocol and the informed consent form. The approved consent form should clearly reflect the EC approval date.

Failure to obtain a signed and hand dated informed consent prior to the procedure constitutes a protocol violation, which is reportable to the EC.

**3.5 Baseline Procedures**

Ambulatory patients who failed standard triple first line H. pylori eradication therapy will be approached by a member of the study research team. A study research staff member will explain the purpose, procedures and intent of the study to each potential participant. Interested subjects will be invited to join the study and asked to provide a written informed consent prior to initiation of any study-related procedure.

*Enrollment*

Subjects who failed to meet the clinical inclusion and exclusion criteria will not be included in the study. Eligible subjects will be randomized to one of the two treatment arms in a1:1 ratio sequential therapy and quadruple therapy, by blocks of four.

*7 days after completing treatment*

First evaluation will take place one week after completing the treatment. A study research staff member will ask each patient about the compliance and adverse effect .

*4 weeks after completing treatment*

Starting with Four weeks after completing treatment and till 16 week C-urea breath test will be performed

*8 weeks after completing treatment*

Eight weeks after completing treatment the results of C-urea breath will be checked

**3.6 Data collection**

Research coordinator at the clinical site will perform primary data collection from source document (e.g., hospital chart, office record) reviews. Data will be entered by the site personnel into eCRFs on the internet-based Electronic Data Capturing (EDC) system. Data will be recorded via a secured web form and stored centrally in a relational database management system to be developed for this purpose. All sites will have password protected access to the web-based system to record data either directly or from paper-based forms. The web system will not include names or the national ID numbers; patients will be identified through a unique identifier assigned for the study purposes. Names and ID numbers will be stored on a separate system, inaccessible from the Internet.

1  Suerbaum S, Michetti P. Helicobacter pylori infection. N Engl J Med 2002;347:1175–86.

2  Vergara M, Vallve M, Gisbert JP, Calvet X. Meta-analysis: comparative efficacy of different proton-pump inhibitors in triple therapy for Helicobacter pylori eradication. Aliment Pharmacol Ther 2003;18:647–54.

3  Hojo M, Miwa H, Nagahara A, Sato N. Pooled analysis on the efficacy of the second-line treatment regimens for Helicobacter pylori infection. Scand J Gastroenterol 2001;36:690–700.

4  Malfertheiner P, Megraud F, O'Morain C, et al. Current concepts in the management of Helicobacter pylori infection – the Maastricht 2–2000 Consensus Report. Aliment Pharmacol Ther 2002;16:167–80.

5  Malfertheiner P, Megraud F, O'Morain C, et al. Current concepts in the management of Helicobacter pylori infection – The Maastricht III Consensus Report. Gut 2007;56:772–81.

6 Lee JH, Cheon JH, Park MJ, et al. The trend of eradication rates of second-line quadruple therapy containing metronidazole for Helicobacter pylori infection: an analysis of recent eight years. Korean J Gastroenterol 2005;46:94–8.

7  Houben MH, van de Beek D, Hensen EF, Craen AJ, Rauws EA, Tytgat GN. A systematic review of Helicobacter pylori eradication therapy – the impact of antimicrobial resistance on eradication rates. Aliment Pharmacol Ther 1999;13:1047–55.

8  Megraud F, Lamouliatte H. Review article. the treatment of refractory Helicobacter pylori infection. Aliment Pharmacol Ther 2003;17:1333–43

9  Megraud F. H. pylori antibiotic resistance: prevalence, importance, and advances in testing. Gut 2004;53:1374–84

10  Graham DY. Therapy of Helicobacter pylori: current status and issues. Gastroenterology 2000;118:S2–8.

11  Dore MP, Leandro G, Realdi G, Sepulveda AR, Graham DY. Effect of pretreatment antibiotic resistance to metronidazole and clarithromycin on outcome of Helicobacter pylori therapy: a meta-analytical approach. Dig Dis Sci 2000;45:68–76.

12  Kim JM. Antibiotic resistance of Helicobacter pylori isolated from Korean patients. Korean J Gastroenterol 2006;47:337–49.

13  Kim N, Kim JM, Kim CH, et al. Institutional difference of antibiotic resistance of Helicobacter pylori strains in Korea. J Clin Gastroenterol 2006;40:683–7

14  Keating GM, Scott LJ. Moxifloxacin: a review of its use in the management of bacterial infections. Drugs 2004;64:2347–77.

15  Di Caro S, Ojetti V, Zocco MA, et al. Mono, dual and triple moxifloxacin-based therapies for Helicobacter pylori eradication. Aliment Pharmacol Ther 2002;16:527–32.

16  Nista EC, Candelli M, Zocco MA, et al. Moxifloxacin-based strategies for first-line treatment of Helicobacter pylori infection. Aliment Pharmacol Ther 2005;21:1241–7.

17  Cheon JH, Kim N, Lee DH, et al. Efficacy of moxifloxacin-based triple therapy as second-line treatment for Helicobacter pylori infection. Helicobacter 2006;11:46–51.

18. Niv et al j. clin gastro-2003, Shmuely et al. mol Nutr Food Res-2002, Samra et al 2002-j.Antimicrobial Chemotherapy. Yahav j.2006 Dagn.Microbiol. Infec.Dis

.

19 H. pylori treeatment-Nimish Vakil, MD, The American journal of Gastroenterology-volume 104, January 2009

20 L.Gatta, MD, Sequential therapy or triple therapy for h.pylori infection. Systematic review and meta-analysis of randomized controlled trials in adults and children , Am. J.Gastroenterol. -2009;104:3069-3079

21 de Francesco V, Margiiotta M, Zullo A et al. Clarithromycin resistant genotypes and eradication of H.pylori –Ann. Intern. Med. 2006; 144; 94-100
